# Values and implicit self-stigmatization among people with mental disorders

**DOI:** 10.1192/j.eurpsy.2021.1439

**Published:** 2021-08-13

**Authors:** G. Arina, I. Oleichik, E. Fyodorova, M. Iosifyan

**Affiliations:** 1 Psychology, Lomonosov Moscow State University, Moscow, Russian Federation; 2 Clinical Department Of Endogenous Mental Disorders And Affective States, Mental Health Research Center of Russian Academy of Medical Sciences, Moscow, Russian Federation; 3 Psychology, University of St Andrews, School of Divinity, St Andrews, United Kingdom

**Keywords:** values, self-stigmatization, implicit self-stigmatization, schizophrénia

## Abstract

**Introduction:**

People with mental disorder can share negative stereotypes, related to mental disorders. This might cause self-stigmatization, which is negatively related to quality of life and compliance with treatment. This self-stigmatization can be non-conscious or implicit, which might complicate it detection and further therapy.

**Objectives:**

In present study we investigated the role of values in implicit self-stigmatization among 40 women diagnosed with schizophrenia (mean age 23.77 years ±6).

**Methods:**

Participants completed the Portrait Value Questionnaire (Schwartz, 2003) and two brief implicit association tests (BIAT), measuring implicit self-esteem and attitudes towards mental disorders (Corrigan et al., 2010). The results of two BIATs were combined as a measure of implicit self-stigmatization.

**Results:**

A linear regression model was built. Four values (self-enhancement, self-transcendence, openness to change and conservation values) were entered as independent variables, while implicit self-stigmatization – as dependent variable. It was found that self-transcendence values were marginally negatively related to implicit self-stigmatization (b=-.122, β=-.398, SE=.064, p=.067), while other values were not significantly related to it (ps>.125).

**Conclusions:**

Self-transcendence values – values related to the well-being of others, which include tolerance, altruism and protection for the welfare of all people and for nature – are negatively related to implicit or non-conscious self-stigmatization. This finding, although marginally significant, is in line with previous studies. Previous studies showed that self-transcendence values are also negatively associated with explicit or conscious self-stigmatization (Lannin et al., 2020). Thus, these values can be targets for programs which aim to decrease self-stigmatization tendencies among patients with mental illness.

